# Combining Machine Learning with Metabolomic and Embryologic Data Improves Embryo Implantation Prediction

**DOI:** 10.1007/s43032-022-01071-1

**Published:** 2022-09-12

**Authors:** Aswathi Cheredath, Shubhashree Uppangala, Asha C. S, Ameya Jijo, Vani Lakshmi R, Pratap Kumar, David Joseph, Nagana Gowda G.A, Guruprasad Kalthur, Satish Kumar Adiga

**Affiliations:** 1 Division of Clinical Embryology, Department of Reproductive Science, Kasturba Medical College, Manipal Academy of Higher Education, Manipal, 576 104 India; 2 Division of Reproductive Genetics, Department of Reproductive Science, Kasturba Medical College, Manipal Academy of Higher Education, Manipal, 576 104 India; 3 Department of Mechatronics Engineering, Manipal Institute of Technology, Manipal Academy of Higher Education, Manipal, 576 104 India; 4 Department of Data Science, Prasanna School of Public Health, Manipal Academy of Higher Education, Manipal, 576 104 India; 5 Department of Reproductive Medicine and Surgery, Kasturba Medical College, Manipal Academy of Higher Education, Manipal, 576 104 India; 6 NMR Research Centre, Indian Institute of Science, Bangalore, 560 012 India; 7 Northwest Metabolomics Research Center, Mitochondria and Metabolism Center, Anesthesiology and Pain Medicine, University of Washington, Seattle, WA USA; 8 Division of Reproductive Biology, Department of Reproductive Science, Kasturba Medical College, Manipal Academy of Higher Education, Manipal, 576 104 India

**Keywords:** ANN, Blastocyst, Machine learning, Metabolomics, NMR spectroscopy

## Abstract

This study investigated whether combining metabolomic and embryologic data with machine learning (ML) models improve the prediction of embryo implantation potential. In this prospective cohort study, infertile couples (*n*=56) undergoing day-5 single blastocyst transfer between February 2019 and August 2021 were included. After day-5 single blastocyst transfer, spent culture medium (SCM) was subjected to metabolite analysis using nuclear magnetic resonance (NMR) spectroscopy. Derived metabolite levels and embryologic parameters between successfully implanted and failed groups were incorporated into ML models to explore their predictive potential regarding embryo implantation. The SCM of blastocysts that resulted in successful embryo implantation had significantly lower pyruvate (*p*<0.05) and threonine (*p*<0.05) levels compared to medium control but not compared to SCM related to embryos that failed to implant. Notably, the prediction accuracy increased when classical ML algorithms were combined with metabolomic and embryologic data. Specifically, the custom artificial neural network (ANN) model with regularized parameters for metabolomic data provided 100% accuracy, indicating the efficiency in predicting implantation potential. Hence, combining ML models (specifically, custom ANN) with metabolomic and embryologic data improves the prediction of embryo implantation potential. The approach could potentially be used to derive clinical benefits for patients in real-time.

**Supplementary Information:**

The online version contains supplementary material available at 10.1007/s43032-022-01071-1.

## Introduction

Embryo morphology is independent of many factors that play crucial roles in embryo viability [1–5]. Despite its known limitations, assessing embryo morphology remains the standard approach for embryo quality assessment [6, 7]. To overcome these limitations, new techniques such as time-lapse imaging, metabolomics, and preimplantation genetic testing for aneuploidy (PGT-A) are being evaluated as alternative approaches for predicting embryo implantation potential [6].

Biomarkers derived from a metabolomics approach have shown contradictory results regarding predicting embryo viability and pregnancy outcomes [8–13]. Further, there is no conclusive evidence that embryo metabolomic data alone can significantly improve the prediction of assisted reproductive technology (ART) outcomes [8, 14]. Hence, there is a continued search for tools that can accurately assess embryo implantation potential alone or in conjunction with other non-invasive methods.

Artificial intelligence (AI)–based models outdo human learning and decision-making even with limited sample sizes [15, 16]. In ART, AI-based analysis combined with patient characteristics, embryo morphokinetics, or embryo microscopic image analysis has been used to predict implantation and pregnancy outcomes [17–21]. The combination of “omics” technology and machine learning (ML) has been suggested to be able to improve ART outcome prediction [22]. A recent study demonstrated that combining a deep learning model with day-3 metabolite profiles predicted blastocyst development [23]. However, we believe that an accurate prediction of implantation potential has a higher clinical value than that of blastulation. Therefore, our approach in this study was to explore the possibility of incorporating metabolomic profiles of human blastocyst spent culture medium (SCM) and embryologic data into ML models to enhance the accuracy of embryo implantation prediction in patients undergoing single blastocyst transfer cycles.

## Materials and Methods

### Patient Selection

This prospective study included 56 couples undergoing ART at a university infertility clinic between February 2019 to August 2021. The study was initiated after obtaining approval from the Institutional Ethics Committee (Ref. 429/2019). Written informed consent was obtained from all study participants. Patients fulfilling the following criteria were included in this study: (i) women less than 35 years of age having regular menstrual cycles; (ii) no medical history of surgery or any abnormalities diagnosed related to reproductive organs; (iii) absence of conditions such as endometriosis, adenomyosis, tubal abnormalities, uterine myoma, and other metabolic/endocrinological diseases, such as hypo/hyperthyroidism or hyperprolactinemia; (iv) the male partners with semen characteristics above the WHO 2010 reference range. In addition, only couples undergoing intracytoplasmic sperm injection followed by day-5 single embryo transfer were included in this study. Patient information, including demographic characteristics and data from routine clinical investigations, is presented in Table 1.

**Table 1:** Patient’s demographics and clinical characteristics

Age-female (year±SEM)	32.96 ± 0.63
Age-male (year±SEM)	37.79 ± 0.71
Duration of infertility (year ±SEM)	5.89 ± 0.37
Basal FSH (mIU/mL±SEM)	6.39 ± 0.31
Basal LH (mIU/mL±SEM)	5.91 ± 0.47
Basal E2 (pg/mL±SEM)	40.82 ± 2.57
AMH (ng/mL±SEM)	3.63 ± 0.33
AFC (n±SEM)	15.86 ±1.1
Length of stimulation (days ±SEM)	9.54± 0.19
Estradiol (pg/mL±SEM) on the day of trigger	3464.27± 249.91
LH (mIU/mL±SEM)	2. 73 ± 0.32
Progesterone (ng/mL±SEM)	0.94 ± 0.11
Endometrial thickness (mm±SEM)	10.06 ±0.32
Oocyte maturation rate (%± SEM)	91.61 ± 1.40
Sperm concentration (millions/mL±SEM)	48.47 ± 5.39
Sperm total motility (%± SEM)	47.26 ± 3.23
Sperm morphology (%± SEM)	23.52 ± 2.17
Sperm DNA fragmentation index (%± SEM)	9.01 ± 0.85
Fertilization rate (%± SEM)	76.72 ± 2.91
Blastocyst rate (%± SEM)	73.77 ± 5.05

### Controlled Ovarian Stimulation (COS) and Oocyte Aspiration

An antagonist protocol was used for COS. Briefly, recombinant follicle-stimulating hormone (rFSH; Gonal F®; Merck Biopharma), with a dose ranging from 225 to 450 IU/day based on age, was administered from the second day of the menstrual cycle, and anti-Müllerian hormone (AMH) level and antral follicular count (AFC) were assessed. Subsequently, rFSH dose adjustment (either increase or decrease) was conducted based on the ovarian response until the day before human chorionic gonadotropin (hCG) administration. Pituitary downregulation was achieved by administering a gonadotropin-releasing hormone (GnRH) antagonist (Citrotide® 0.25 mg; Merck Biopharma) from day 5 of COS. Recombinant hCG (Ovitrelle® 250 mg; Merck Biopharma) was used to trigger the final oocyte maturation when at least four follicles reached a mean diameter of 18 mm. Oocyte cumulus complexes were collected via the ultrasound-guided transvaginal route, rinsed, and placed in ONESTEP medium (#V-OSM-20; Vitromed GmbH, Germany) at 37°C in 6% CO_2_ for 2–3 h until enzymatic denudation.

### Fertilization and Embryo Evaluation

Intracytoplasmic sperm injection was used to fertilize mature (metaphase II) oocytes. Injected oocytes were then washed and cultured individually in a 30-μL droplet of ONESTEP medium overlaid with oil (#V-OIL-P100; Vitromed GmbH, Germany) at 37°C, 6% CO_2_, and 5% O_2_ in a MIRI® Multiroom incubator (ESCO Medical, Singapore). Fertilization was assessed at 16–18 h after the intracytoplasmic sperm injection. Embryos were evaluated on day 3 and day 5 of development as per the European Society of Human Reproduction and Embryology (ESHRE) consensus [24]. On day 5, only one top-quality blastocyst (3, 1, 1 or 4, 1, 1) was selected for transfer. If the fresh transfer was not performed, embryos were cryopreserved by vitrification for subsequent transfer.

SCM samples from transferred/frozen blastocysts (*n*=56) along with medium control samples (droplets of ONESTEP medium without an embryo) (*n*=44) were carefully collected without oil contamination, and 25 μL of each was placed into a labeled sterile cryovial, snap-frozen in liquid nitrogen, and stored at −80°C until nuclear magnetic resonance (NMR) spectroscopic analysis.

### NMR Sample Preparation and Analysis

A dilution solution was prepared using D_2_O (deuterium oxide), with TSP (sodium salt of 2,2,3,3 tetradeutero-3-(trimethylsilyl propionate) as the standard reference compound; 0.05 g TSP/mL D_2_O was diluted by a factor of 10 using D_2_O. After thawing the SCM and medium control samples at room temperature, 25 μL was diluted with a 10 μL dilution solution. The mixture was then transferred to 1.7-mm NMR tubes. Thus, all the metabolites present in the samples were diluted up to 1.4 times with the dilution solution.

NMR experiments were carried out using a Bruker 800-MHz AVANCE III NMR spectrometer (Bruker Biospin Ag, Fällanden, Switzerland) equipped with a 1.7-mm cryo-probe at 298 K. One-dimensional (1D) ^1^H NMR spectra were obtained using the Carr-Purcell-Meiboom-Gill (CPMG) pulse sequence. A CPMG 180° pulse train for a duration of 12 ms was used to suppress residual protein signals from the media. Each spectrum was obtained using 9615-Hz spectral width, 5-s relaxation delay, 16-k time domain points, 4 dummy scans, and 256 transients. The time domain data (free induction decay) were apodized with a shifted sine bell window function (SSB = 2) and zero-filled to 65536 points prior to Fourier transformation. TopSpin v3.6.2 (Bruker) was used for NMR data acquisition and processing.

A total of 100 1D ^1^H spectra were acquired, comprising spectra related to the SCM of the embryos (*n*=56) and medium control samples (*n*=44). Based on the human metabolome database [25, 26], 13 metabolite peaks were identified: nine amino acid metabolites (leucine, Leu; isoleucine, Ile; valine, Val; methionine, Met; threonine, Thr; lysine, Lys; tyrosine, Tyr; histidine, His; phenylalanine, Phe) and four carbohydrate and metabolic intermediates (pyruvate, Pyr; lactate, Lac; citrate, Cit; glucose, Glc). Relative concentrations of the identified metabolites were then determined by normalizing the metabolite peak integrals to the peak integral of the internal standard, TSP. Further region-wise integration was performed with “intser” in TopSpin v3.6.2; each spectrum was divided into 30 integral regions.

### ML Model Training and Testing Procedures

**Fig. 1:**
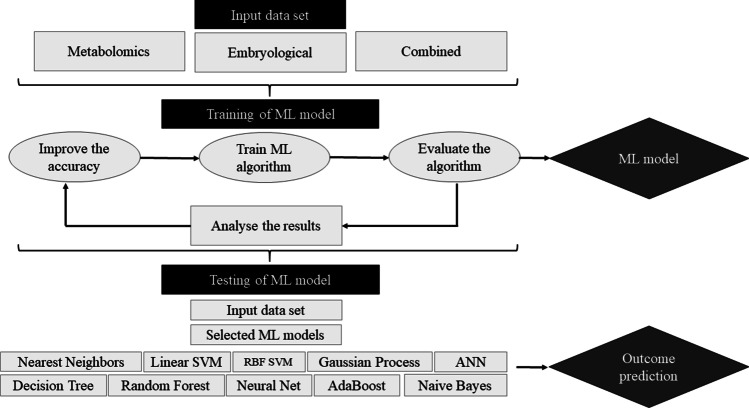
Flowchart of the machine learning (ML) model training and testing procedures

A flowchart of the ML model training and testing procedures is shown in Fig. 1. In order to compare the performance of classical ML programs, several well-known ML algorithms were considered. Nearest neighbors, linear support vector machine (SVM), radial basis function (RBF) SVM, gaussian process, decision tree, random forest, neural net, AdaBoost, and naïve Bayes were used and then compared to custom artificial neural network (ANN)–based binary classification models. As the above classical ML models have an overfitting issue, a custom ANN model was incorporated to provide a better prediction with weight regularization. The samples were randomly divided into two groups: the training set constituted 80% samples (which was used to train the models to predict embryo implantation potential) and the testing set constituted 20% samples (which was used to check and validate the performance of the models).

### Input and Output Data

Prediction models were constructed using three sets of data: (i) SCM metabolites identified by NMR spectroscopy; (ii) oocyte and embryologic characteristics such as number of matured oocytes retrieved, maturation rate, fertilization rate, number of nucleolar precursor bodies (NPBs) observed in the zygote, number of embryos progressed to day 3, blastocyst rate and quality (on day 5), and the grade of the embryo preferred for the transfer (on day 3 and day 5); and (iii) various combinations of metabolites and oocyte/embryologic characteristics (selecting metabolites based on their roles in different metabolic pathways). Further, each combination involved oocyte and embryologic characteristics along with the following combination of metabolites: combination 1, Glc, Pyr, and Lac; combination 2, Glc, Pyr, and Cit; combination 3, Phe and Tyr; combination 4, Pyr, Cit, Lys, and Thr; combination 5, Glc, Pyr, Thr, Met, and Ile; and combination 6, Glc, Pyr, Cit, Ile, Leu, and Val. The exact parameters involved in each dataset are given in supplementary Table 1. The output data comprised the implantation potential of the individually transferred blastocysts. The input data were preprocessed and transformed to the same scale. The features involved both numeric and nonnumeric data. Nonnumeric data were converted to numeric data and then normalized to obtain values in a similar range.

### Data Classification Using Custom ANN

The custom ANN was built using a sequential model. The variables were first initialized, after which layers were added using the dense functionality, forming the layout of the model. Subsequently, procedures involving a loss function, an Adam optimizer, and metrics (to assess model performance) were conducted. Data on 56 SCM samples were used, with 44 (80%) being used to train the model and 12 (20%) being used to test it. The model was trained using the training data for 50 epochs. Epochs refer to the number of times that the custom. ANN goes through the training data. The model parameters are noted in Supplementary Table 2. The first layer consisted of 30 or 50 neurons, with a rectified linear unit (ReLU) as the activation function followed by a single neuron with a sigmoid activation function. Adam optimizer was used with a learning rate of 0.001 with binary cross-entropy as the loss function. The classical ML model performance was assessed using several metrics, including confusion matrix, receiver operating characteristic (ROC) curve, area under the ROC curve (AUC), and accuracy, whereas accuracy and loss curves are employed in custom ANN for measuring the performance. Typically, the cross-entropy loss is used as loss function for binary classification problems involving ANN models in which the predicted output probability is compared to the actual output. The computed score penalizes the probability-based on the distance from the actual value. The logarithmic penalty yields a small value for a small difference and a large value for a large difference. The objective function involves minimizing the cross-entropy loss, and smaller values represent a better model. A perfect model has a cross-entropy loss of zero. Cross-entropy for a binary or two-class prediction problem is calculated as the mean cross-entropy across all examples. The custom ANN model was run with a batch size of 8 and a total number of 50 epochs. A similar procedure was conducted for each dataset (i.e., the metabolites, embryologic, and combination datasets).

### Software

Data analysis was implemented using https://colab.research.google.com, with TensorFlow, Keras, Sklearn, and NumPy library available in Python v3.7. The plots were created using the Matplotlib library.

### Statistical Analysis

The participants’ demographic and clinical data are presented as mean ± standard error of the Mean (SEM). Statistical differences in metabolite levels between SCM and medium control samples were assessed by independent-sample *t* tests. Statistical differences in metabolite levels among SCM samples from blastocysts that resulted in successful embryo implantation, SCM samples from blastocysts that resulted in embryos that failed to implant, and medium control samples were assessed by repeated-measure analysis of variance (ANOVA) followed by post hoc Tukey’s tests in Jamovi v1.8.1 [27]. Principal component (PC) analysis was carried out in CRAN R v4.0 [28], to explore metabolic differences based on 30 integral regions of 1D ^1^H spectra from samples in the three groups. A two-dimensional bi-plot visualized the first two PCs (PC_1_ and PC_2_), which accounted for 99.61% of the variability in the data. The level of significance was set at <0.05 throughout the study.

## Results

### Patient Characteristics and Embryo Implantation Outcomes

This prospective study included 56 infertile couples who underwent a single day-5 blastocyst transfer during their ART cycle. Patient demographic and clinical characteristics are summarized in Table 1. Notably, only one top-quality day-5 blastocyst was used for transfer. The endometrial thickness in patients was 10.06 ±0.32 mm. In cases involving frozen embryo transfer cycles, patients were followed up until frozen embryo transfer. Implantation was considered successful when the beta hCG level was >100 mIU/mL on day 14 post embryo transfer. Out of the 56 patients, 23 had successful embryo implantation, and 33 had embryos that failed to implant. The implantation rate was 41%.

#### Variation in Relative Levels of Metabolites in SCM

**Fig. 2:**
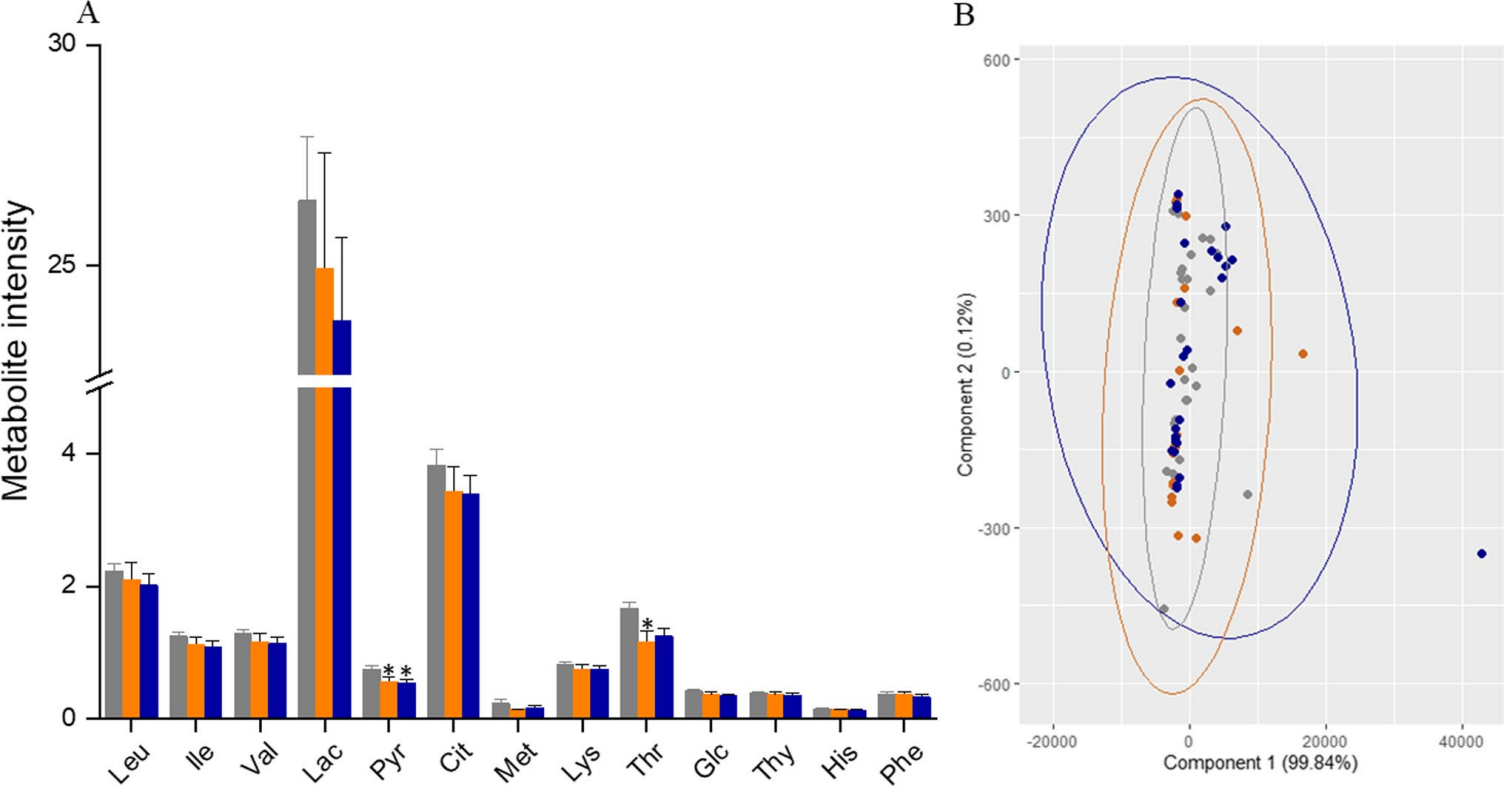
**A** Comparison of the metabolite levels in spent culture medium (SCM) samples from successfully implanted embryos (*n*=23) and embryos that failed to implant (*n*=33) relative to the levels in the medium control samples (*n*=44). B) Principal component analysis (bi-plot) of the region-wise integrals of the three groups. Gray  represents medium control, orange  represents SCM from successfully implanted embryos, and blue  represents SCM from embryos that failed to implant

To understand metabolite utilization by the blastocysts, metabolite levels were compared (i) between SCM and medium control samples, (ii) between SCM samples from successfully implanted embryos and medium control samples and between SCM samples from embryos that failed to implant and medium control samples, and (iii) between SCM samples from successfully implanted embryos and SCM samples from embryos that failed to implant. Supplementary Fig. 1 depicts a representative 1D ^1^H NMR spectrum of ONESTEP medium with peak assignment. Significant reductions in the pyruvate (*p*<0.001) and threonine (*p*<0.002) levels were observed in SCM samples relative to medium control samples (Table 2), indicating that the embryos utilized the metabolites from the culture media. Although similar trends were observed in other metabolites, the differences were not significant. Further, there was a significant difference in the pyruvate level (relative to medium control) for SCM from both successfully implanted embryos (*p*<0.05) and embryos that failed to implant (*p*<0.001), and in the threonine level for SCM from successfully implanted embryos (*p*<0.05). Of note, statistical significance was not demonstrated in relative metabolite levels between the successful and failed implantation groups (Fig. 2A and Table 2).

**Table 2:** Comparison of the relative concentration of metabolites (normalized to TSP) across the study groups with the medium control

Metabolites	Relative concentration (mean± SEM)			
	Medium control (*n*=44)	SCM samples (*n*=56)	Successful implantation (*n*=23)	Failed implantation (*n*=33)
Leucine	2.2 ± 0.12	2.1 ± 0.15	2.1 ± 0.26	2.0 ± 0.17
Isoleucine	1.2 ± 0.07	1.1 ± 0.07	1.1 ± 0.12	1.1 ± 0.09
Valine	1.3 ± 0.07	1.2 ± 0.07	1.2 ± 0.12	1.1 ± 0.09
Lactate	26.5 ± 1.45	24.2 ± 1.54	24.9 ± 2.62	23.7 ± 1.89
Pyruvate	0.8 ± 0.04	0.6 ± 0.04**	0.6 ± 0.06*	0.5 ± 0.05*
Citrate	3.8 ± 0.24	3.4 ± 0.23	3.4 ± 0.37	3.4 ± 0.29
Methionine	0.2 ± 0.06	0.2 ± 0.02	0.1 ± 0.01	0.2 ± 0.03
Lysine	0.8 ± 0.04	0.7 ± 0.05	0.7 ± 0.08	0.7 ± 0.06
Threonine	1.7 ± 0.10	1.2 ± 0.10**	1.2 ± 0.17*	1.3 ± 0.12
Glucose	0.4 ± 0.02	0.4 ± 0.02	0.4 ± 0.04	0.3 ± 0.03
Tyrosine	0.4 ± 0.02	0.4 ± 0.02	0.4 ± 0.04	0.4 ± 0.03
Histidine	0.2 ± 0.01	0.1 ± 0.01	0.1 ± 0.02	0.1 ± 0.01
Phenylalanine	0.4 ± 0.02	0.3 ± 0.02	0.4 ± 0.04	0.3 ± 0.03

** *p* < 0.005 and *p < 0.05, vs medium control

To explore the differences in the unidentified metabolites in the NMR profiles, each spectrum was divided into 30 integral regions. PC analysis of the 30 integral regions (based on 100 samples from 56 patients) was used to explore the variance among the three groups. Fig. 2B shows the resulting two-dimensional PC bi-plot of PC_1_ vs PC_2_, with overlapping data points from three groups which accounted for 99.61% of the variability in the data. There were no identifiable differences in SCM metabolites (relative to medium control) between the implanted and failed embryos (Fig. 2B). Overall, using only SCM metabolite levels determined by NMR spectroscopy did not successfully discriminate among embryos based on their implantation potential.

### Use of ML Models in Predicting Embryo Implantation Potential

**Fig. 3:**
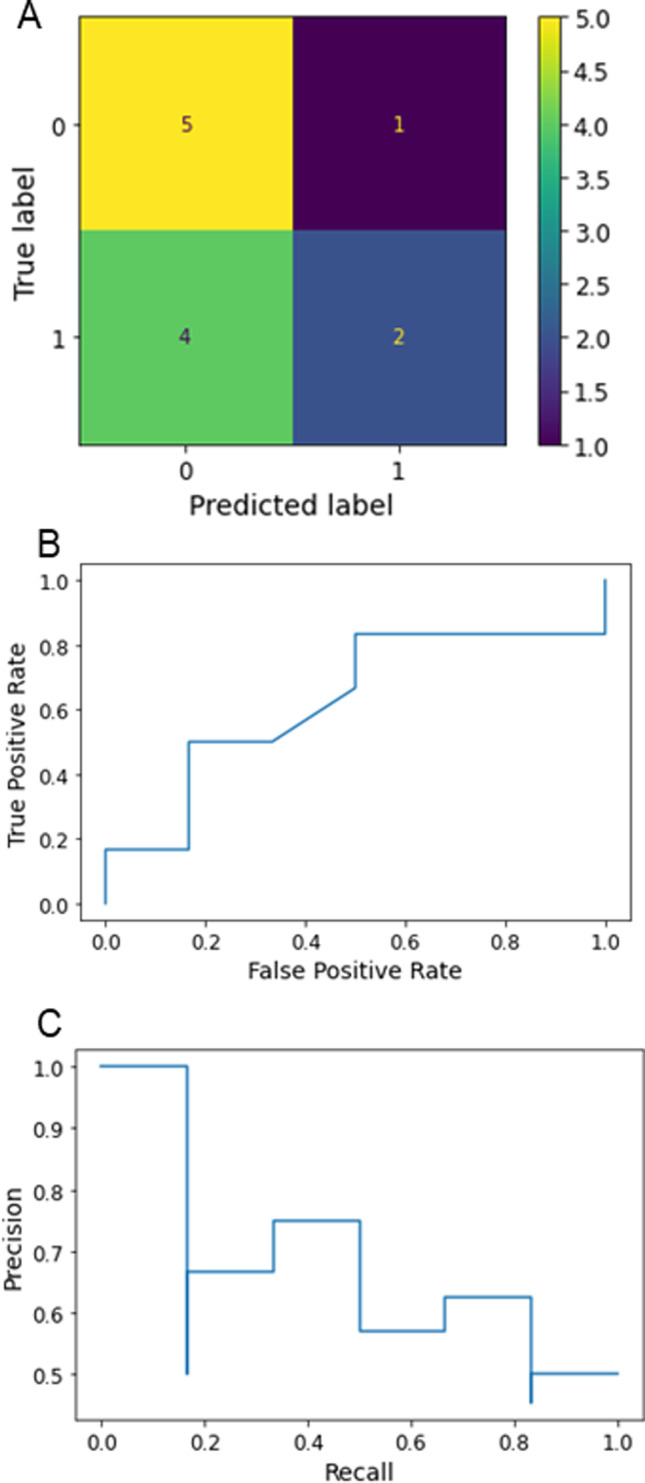
Performance evaluation of classical ML model (random forest) combined with metabolite dataset**. A** Confusion matrix for random forest classifier, **B** receiver operating characteristic (ROC) curve, and **C** precision-recall curve

Initially, classical ML models (nearest neighbors, linear SVM, RBF SVM, gaussian process, decision tree, random forest, neural net, AdaBoost, and naïve Bayes) alone or in conjunction with metabolomic data and/or embryologic data were used to predict the implantation potential of the embryos. Naïve Bayes, AdaBoost, and decision tree performed well when using the metabolite dataset and provided 100% accuracy even with a small dataset (Table 3). Decision tree, random forest, neural net, AdaBoost, and naïve Bayes provided 100% accuracy when using the embryologic data collected from 56 patients (Table 3). However, when combining the metabolomic and embryologic data, the prediction accuracy of all the classical ML models increased, with accuracies of 80–100%. Notably, the combination 3 and 5 datasets provided 100% accuracy in all ML models assessed. The performance of ML model was evaluated based on a confusion matrix, ROC curve, and precision-recall curve (Fig. 3A–C). The confusion matrix provides the details of false positive, false negative, true positive and true negative values. A good classifier is expected to produce a higher true positive and true negative. The classical ML model such as random forest demonstrated poor performance when metabolite data was used (Fig. 3A). In addition, ROC plots the true positive rate against the false-positive rate. For a good classifier, the ROC curve stays away from a linear line. In the sample shown for the traditional random forest model, a poor ROC curve indicates the poor classification of metabolites data (Fig. 3B). Further, the precision-recall rate measures the precision versus recall. The curve shows that random forest has poor capability in classifying the metabolite data (Fig. 3B).

**Table 3:** Accuracy of classical ML-based algorithms for different combination of features

Method	Metabolites	Embryological parameters	Combination of features					
			1	2	3	4	5	6
Nearest neighbors	0.58	0.58	0.92	1.00	1.00	1.00	1.00	1.00
Linear SVM	0.50	0.58	1.00	1.00	1.00	1.00	1.00	1.00
RBF SVM	0.66	0.50	0.83	1.00	1.00	1.00	1.00	1.00
Gaussian process	0.83	0.66	1.00	1.00	1.00	1.00	1.00	1.00
Decision tree	1.00	1.00	1.00	1.00	1.00	1.00	1.00	1.00
Random forest	0.75	1.00	1.00	0.92	1.00	0.83	1.00	0.83
Neural net	0.5	1.00	1.00	1.00	1.00	1.00	1.00	1.00
AdaBoost	1.00	1.00	1.0	1.0	1.0	1.0	1.0	1.0
Naïve Bayes	1.00	1.00	1.0	1.0	1.0	1.0	1.0	1.0

*Combination #1: Glc, Pyr, Lac, oocyte, and embryo parameters*

*# 2: Glc, Pyr, Cit, oocyte, and embryo parameters*

*# 3: Phe, Thy, oocyte, and embryo parameter*

*#4: Pyr, Cit, Lys, Thr, oocyte, and embryo parameters*

*#5: Glc, Pyr, Thr, Met, Iso, oocyte, and embryo parameters*

*#6: Glc, Pyr, Cit, Iso, Leu, Val, oocyte, and embryo parameters*

**Fig. 4:**
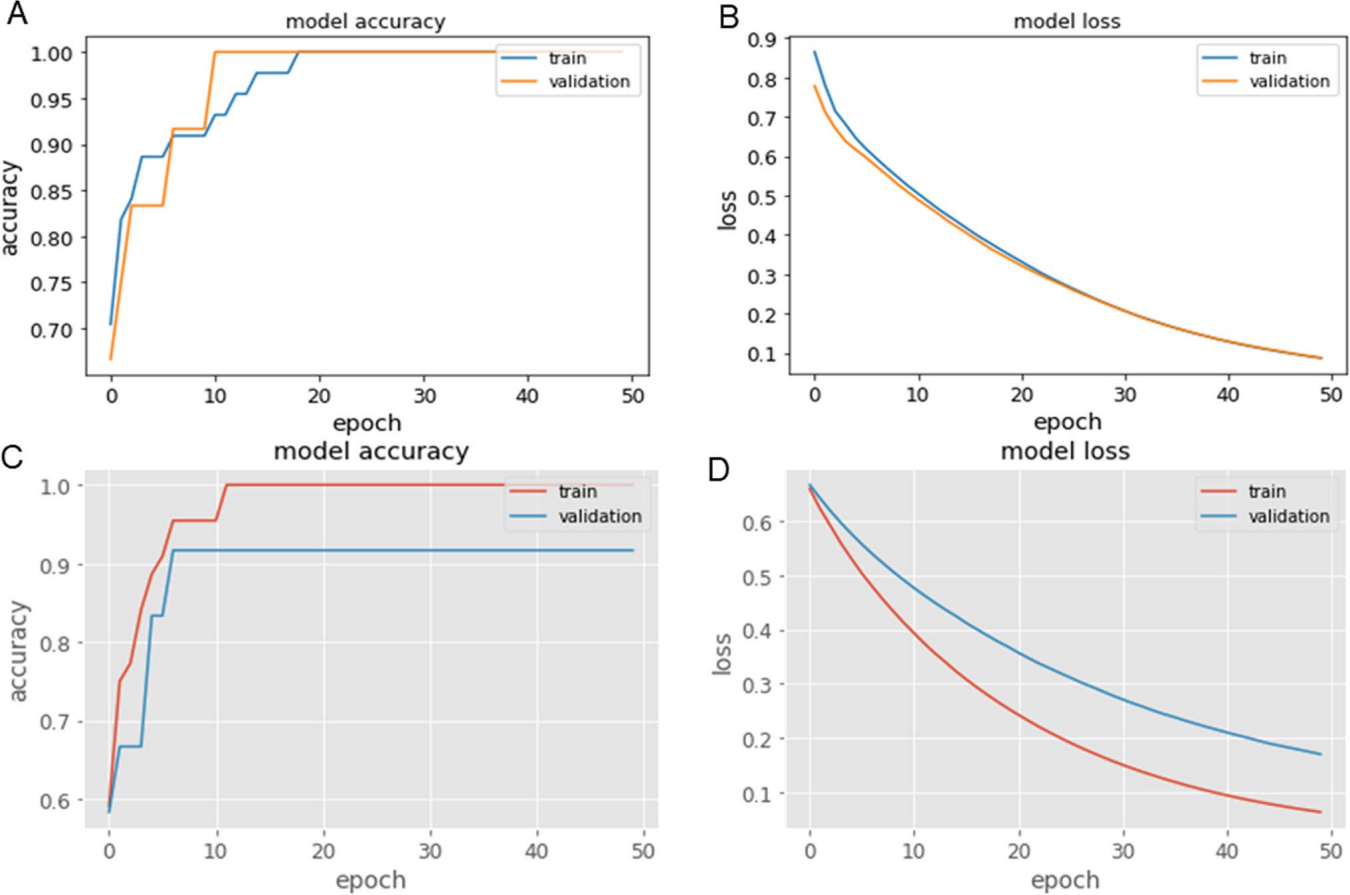
Accuracy and loss curves obtained for the custom ANN model with the training and testing datasets. **A** Accuracy curve (demonstrating 100% accuracy for the training and testing dataset) for ANN model with the metabolite dataset for 50 epochs and **B** loss curve. **C** Accuracy curve for ANN model with embryologic dataset for 30 epochs and **D** loss curve

The custom ANN was also compared with the above state-of-the-art classical ML methods (nearest neighbors, linear SVM, RBF SVM, gaussian process, decision tree, random forest, neural net, AdaBoost, and naïve Bayes). Metabolite data from the NMR peaks (corresponding to 13 metabolites obtained from 56 SCM samples) were used as input data in the custom ANN model. When tested with the training data of 44 and testing data of 12 with the batch size of 8, and number of epochs of 50, the number of neurons present in the first layer was 50, and second layer was 1 with sigmoid activated function. This model had an accuracy of 100% even with a small dataset at lower epochs (Fig. 4A) and a loss of 0.0059 (Fig. 4B). Hence, custom ANN would provide good accuracy if a large dataset was available. Using the similar approach, involving custom ANN and the embryologic dataset (with the training data of 44 and testing data of 12 with the batch size of 8, and number of epochs of 50), the number of neurons present in the first layer was 30 and second layer was 1 with sigmoid activated function produced an accuracy of 91.67% for the testing dataset (Fig. 4C) and a loss of 0.1125 (Fig. 4D). The promising results suggest that custom ANN is very efficient in predicting implantation outcomes based on metabolomic or embryologic data and any of the combinations assessed.

## Discussion

The lack of conclusive evidence on the value of metabolomic biomarkers for predicting ART outcomes prompted us to combine ML models with conventionally used embryological data along with NMR-identified metabolite levels. For the first time, this study incorporated data generated from SCM metabolite analysis into ML models. Interestingly, the data from this study suggests that when classical ML models are used, incorporating both metabolomic and embryologic data significantly improves the prediction accuracy compared to metabolomic data alone. Further, it is clear from our results that custom ANN models predicted the embryo implantation potential with 100% accuracy when utilizing metabolomic features.

The embryo quality and endometrial receptivity are two major determining factors in the embryo implantation process. Our study included only morphologically superior (top-graded) blastocyst on day 5. Since endometrial thickness can influence ART outcome [29, 30], we ensured that the endometrial thickness in our study subjects was comparable between positive (9.67±0.48mm) and negative implantation (10.29±0.44mm; *p*>0.05). In addition, women with adenomyosis and huge uterine myoma were excluded from the study as it can influence the implantation process. Thus, we believe that both embryo and endometrial factors that can influence the embryo implantation process were controlled in our experimental settings.

Metabolomics approaches have been shown to have the potential for identifying biomarkers related to embryo development and thus improving the outcomes of ART cycles [31]. Several NMR-based studies have demonstrated associations between SCM metabolites and implantation/pregnancy outcomes [11, 32, 33]. Specifically, metabolites such as pyruvate, glucose, glutamate, and amino acid turnover have been suggested as biomarkers of embryo development, implantation potential, and clinical pregnancy [11, 12, 33]. Conversely, other studies using NMR spectroscopy as an analytical tool have failed to demonstrate any associations between SCM metabolites and embryo implantation potential [9, 34, 35]. Although pyruvate and threonine levels were significantly altered in SCM from successfully implanted embryos relative to the medium control, we could not establish significant differences between the successful and failed implantation groups. This is in agreement with the recent reports that metabolomics approaches alone could not efficiently enhance ART outcome prediction [8, 14]. Technical variations in SCM sampling, processing, contamination, and analytical complexity are known to affect the results [6, 14, 36, 37]. The differences in the composition of the commercial embryo culture media, culture conditions like oxygen level, culture medium volume, embryonic developmental stage, and the sex of the embryo can also lead to inconsistencies in metabolomic-based studies [6]. Hence, there is a need to combine metabolomics approaches with other approaches to improve the predictive value.

AI-based analysis for predicting ART-related pregnancy outcomes is gaining in popularity. ML is a subtype of AI-based analysis where computer-based algorithms are used to understand the pattern present in a complex set of data and help with prediction. Several ML algorithms such as decision tree, random forest, SVM, and naïve Bayes classifier are being used in reproductive medicine; as reviewed by Wang et al. [38], external validation of van Loendersloot’s model using clinical data alone led to 64.0% accuracy [39]; whereas a naïve Bayes model with embryologic data led to 80.4% accuracy [21]. Age of the female partner, number of embryos formed, and serum E2 level on the day of trigger were identified as the best features to predict outcomes [40], although the overall accuracy was below 85%. Oocyte and embryologic characteristics such as oocyte maturity, fertilization rate, number of nuclear precursor bodies (NPBs), embryo progression to day 3, blastocyst rate and quality (on day 5), and the grade of the embryo preferred for transfer (on day 3 and day 5) were also analyzed as these parameters have demonstrated as predictive factors of embryo development and implantation potential using conventional or AI based analysis [40–46].

Incorporation of AI-based analysis was recently recommended for improving the efficacy of embryo implantation potential prediction by omics-based approaches [22]. A recent study has incorporated proteomic profile of euploid blastocysts and their morphology in AI-based prediction of embryo implantation potential [47]. However, there are no studies exploring the combination of ML models and NMR-derived metabolite data for predicting implantation potential. Recently, a deep learning model combined with Raman profiles generated from day-3 embryos was used to predict blastocyst development [23]. In line with the earlier results utilizing ML models for patient characteristics to predict embryo implantation potential [48–50], certain ML models (such as nearest neighbors, RBF SVM, decision tree, random forest, and neural net) provided accuracies of 50–67% (moderate accuracies) when metabolomic data alone was used. Moreover, when using embryologic data alone, nearest neighbors, RBF SVM, and decision tree provided accuracies of 50–67%. Although we observed 100% accuracy when combining most of the classical ML models with both metabolomic and embryologic data, the results could not be substantiated with the current small dataset, as classical ML models have an overfitting issue. Hence, a custom ANN model was employed to overcome this data issue with the regularization method. ANN models have the ability to model nonlinear and complex data, they can more effectively infer unseen data, and dropout helps to overcome the overfitting issue. Classical ML models were initially used and then compared to the advanced ANN models, which provided more than 90% accuracy for both metabolomic (100%) and embryologic (92%) data with a small sample size. Hence, ANN could be used with complex data to accurately predict outcomes in real time. In addition, ML models should be tested with a large dataset.

The strength of this study is that only day-5 blastocyst transfer cycles were used to assess our combined approach to predicting embryo implantation potential. Even after using a high-resolution (800 MHz) NMR spectrometer equipped with a cryogenically cooled micro-coil (1.7 mm) probe to profile SCM metabolites, it was not possible to obtain a differential metabolite signature between successful and failed implantation groups. Further, classical ML models have an overfitting problem, which may exaggerate the prediction when a small sample size is used, whereas ANN can overcome this issue with an added regularization to the loss function

## Conclusion

The observations made in this study open up the possibility of integrating multiple datasets with ML models to improve the prediction of embryo implantation potential. Combining ML models (specifically ANN models) with metabolomic and embryologic data may improve the prediction of embryo implantation potential. This approach should be tested in large and diverse datasets and it potentially could be used to derive clinical benefits for patients in real time.

## Supplementary information

Supplementary Table 1 (DOCX 14 kb)

Supplementary Table 2 (DOCX 12 kb)

Supplementary Fig. 1 Representative one-dimensional ^1^H NMR spectrum of ONESTEP embryo culture medium used in the study. The figure shows the assignment of peaks for different metabolites. The x-axis represents the chemical shift in parts per million. (PNG 79 kb)

High resolution image (TIF 151 kb)

## Data Availability

The data and material that support the findings of this study are available from the corresponding author upon request.
