# Supplementary material for: Combining Machine Learning with Metabolomic and Embryologic Data Improves Embryo Implantation Prediction
Source: Reprod Sci. 2022 Sep 12;30(3):984–94. doi: 10.1007/s43032-022-01071-1 (PMC10014658; doi:10.1007/s43032-022-01071-1)
Supplement: Supplementary file 1 — (DOCX 14 kb) [file 43032_2022_1071_MOESM1_ESM.docx]

Supplementary table 1: Input dataset used in training the model.

| **Metabolite data set** | **Oocytes and embryo characteristics** |
| --- | --- |
| Leucine | Number of mature oocytes retrieved |
| Isoleucine | Maturation rate |
| Valine | Fertilization rate |
| Lactate | Number of nucleolar precursor bodies in zygote |
| Pyruvate | Total cell number on day 3 |
| Citrate | Embryo grade on day 3 |
| Methionine | Blastocyst rate |
| Lysine | Blastocyst grade on day 5 |
| Threonine |  |
| Glucose |  |
| Tyrosine |  |
| Histidine |  |
| Phenylalanine |  |
