# Supplementary material for: Combining Machine Learning with Metabolomic and Embryologic Data Improves Embryo Implantation Prediction
Source: Reprod Sci. 2022 Sep 12;30(3):984–94. doi: 10.1007/s43032-022-01071-1 (PMC10014658; doi:10.1007/s43032-022-01071-1)
Supplement: Supplementary file 2 — (DOCX 12 kb) [file 43032_2022_1071_MOESM2_ESM.docx]

Supplementary table 2: The model parameters employed for the ANN

Model: "sequential_6"

| **Layer (type)** | **Output Shape** | **Parameter #** |
| --- | --- | --- |
| dense_12 (Dense) | (None, 30) | 450 |
| dense_13 (Dense) | (None, 1) | 31 |

Total parameters: 481

Trainable parameters: 481

Non-trainable parameters: 0
