# Supplementary figures and images for: Combining Machine Learning with Metabolomic and Embryologic Data Improves Embryo Implantation Prediction
Source: Reprod Sci. 2022 Sep 12;30(3):984–94. doi: 10.1007/s43032-022-01071-1 (PMC10014658; doi:10.1007/s43032-022-01071-1)

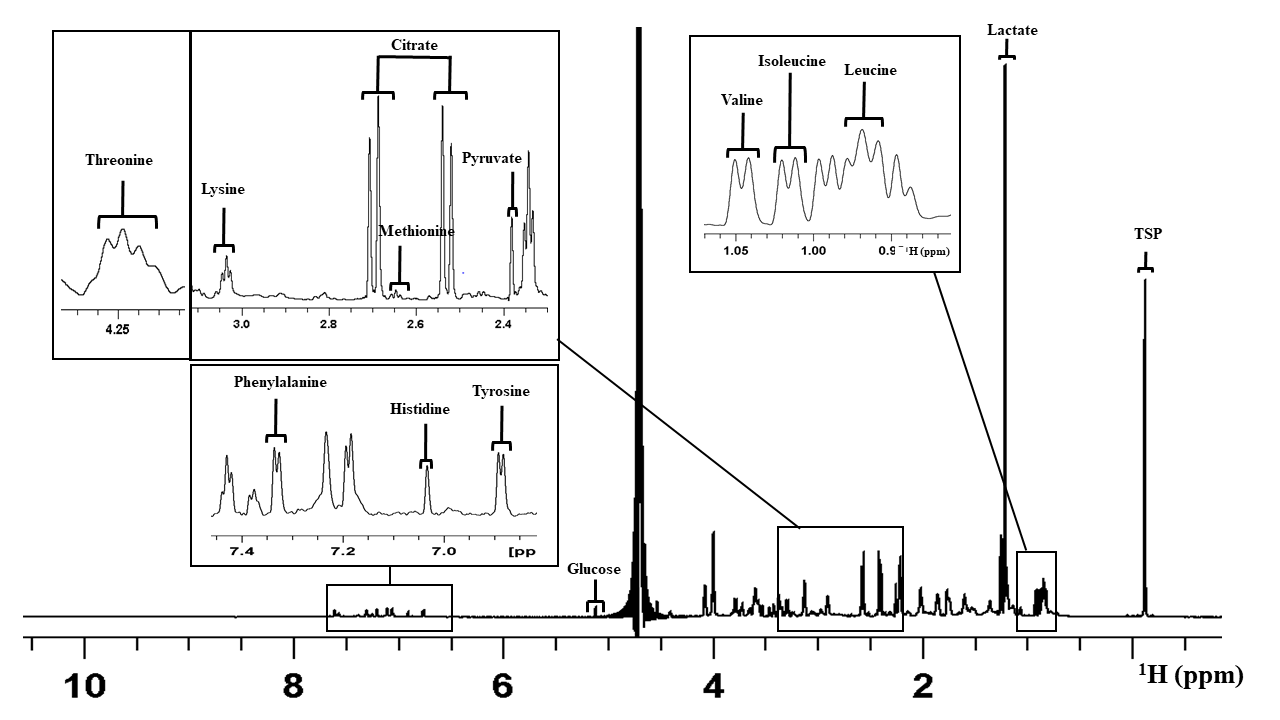

Supplement: Supplementary file 3 — Representative one-dimensional 1H NMR spectrum of ONESTEP embryo culture medium used in the study. The figure shows the assignment of peaks for different metabolites. The x-axis represents the chemical shift in parts per million. (PNG 79 kb) [file 43032_2022_1071_Fig5_ESM.png]

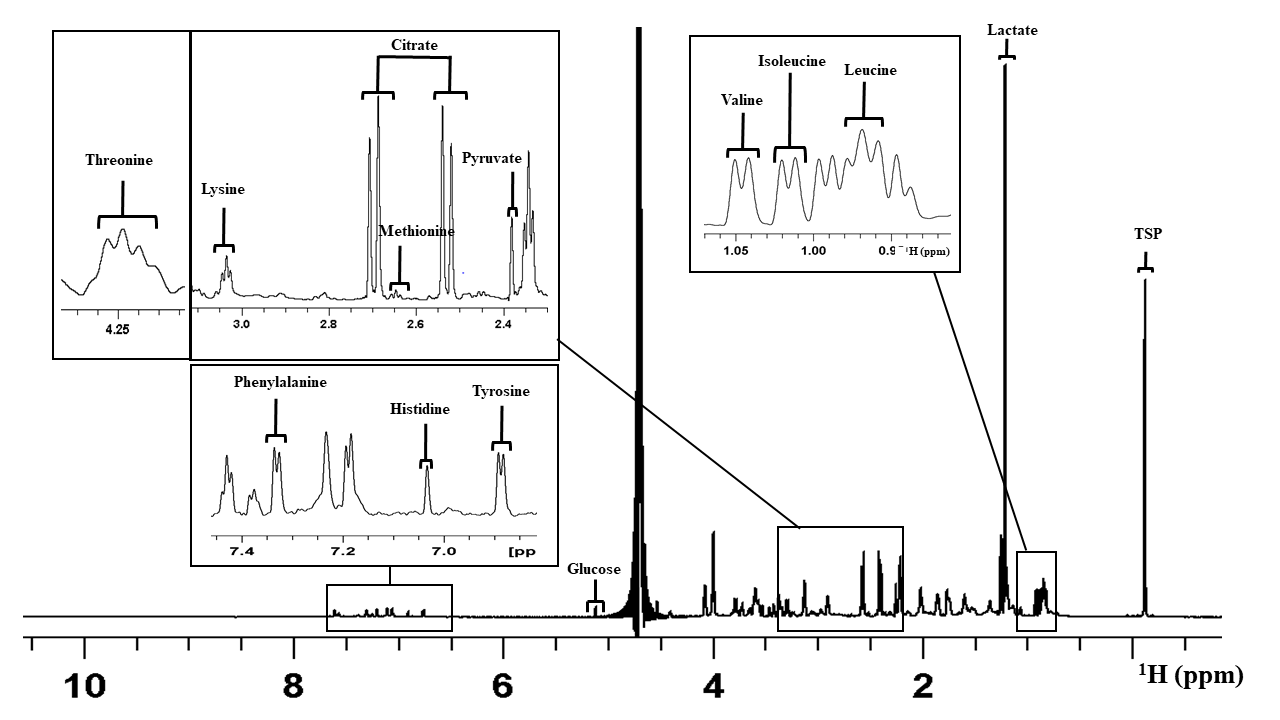

Supplement: Supplementary file 4 — High resolution image (TIF 151 kb) [file 43032_2022_1071_MOESM3_ESM.tif]
